# Supplementary material for: Phylogenetic and Structural Insights into Melatonin Receptors in Plants: Case Study in Capsicum chinense Jacq
Source: Plants (Basel). 2025 Jun 26;14(13):1952. doi: 10.3390/plants14131952 (PMC12252195; doi:10.3390/plants14131952)
Supplement: Supplementary file 1 [file plants-14-01952-s001.zip › supplementaries_submission.pdf]

Supplementary figures

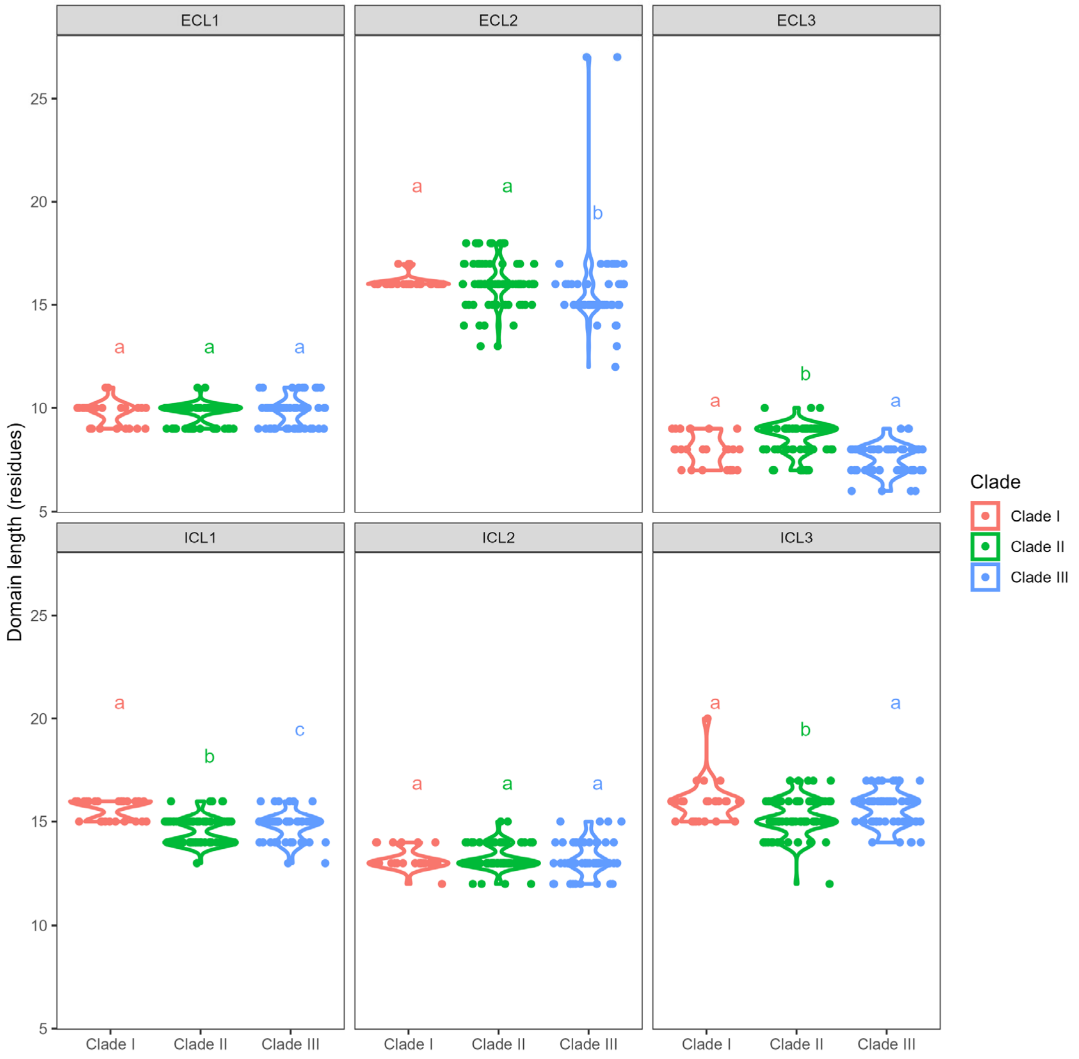

Figure S1: Length variation of extracellular (ECL) and intracellular (ICL) loops in pPMTRs across clades. Violin plots show the distribution of ECL and ICL lengths predicted by Tmbed, with observations displayed as dots. Statistical differences were determined using the Kruskal-Wallis test followed by Dunn's test with the Bonferroni correction ($p < 0.05$)

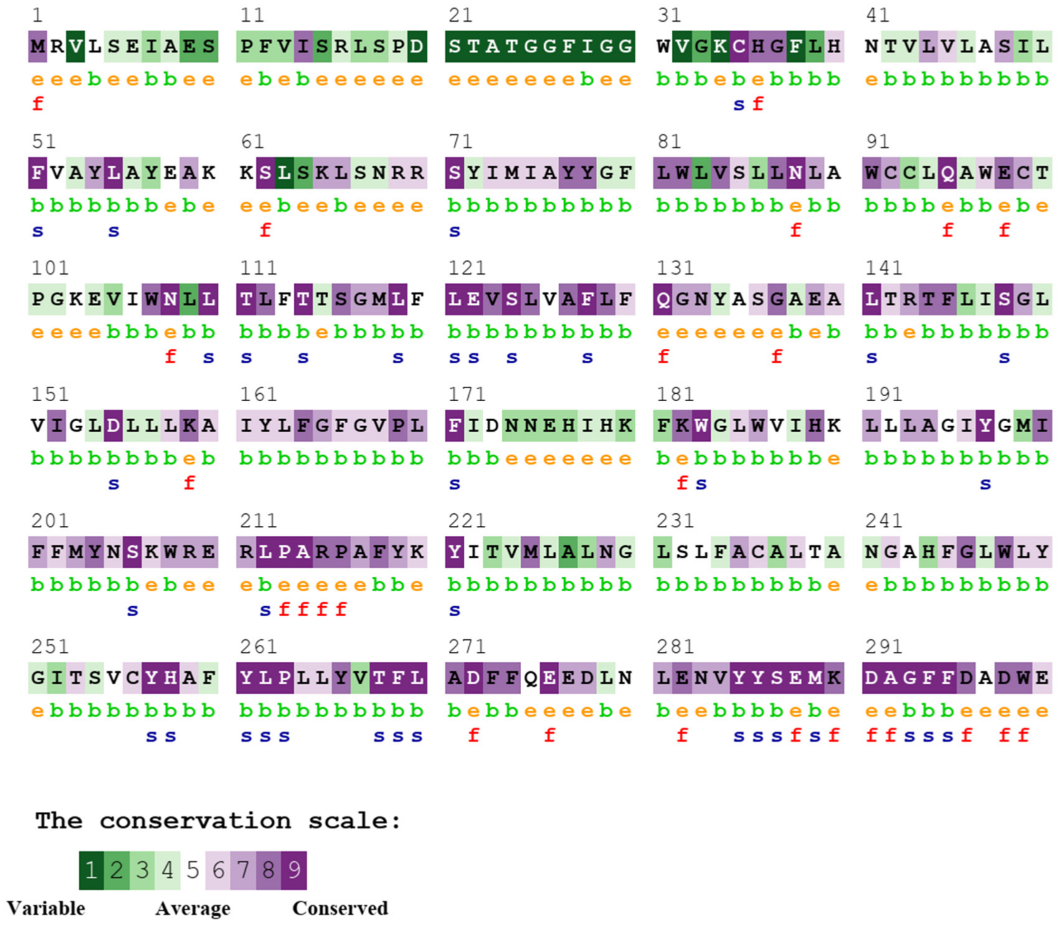

Figure S2: Amino acid conservation of AtPMTR1 homologs. ConSurf web server searched for AtPMTR1 homologues, clustered them based on sequence similarity and selected a representative set. Then these sequences were aligned and the MSA was used for Rate4Site (Pupko et al. 2002) to calculate the evolutionary rate per site. Per-site scores range from one, highly variable (fastest rate), to nine, highly conserved (slowest rate). Residue labels: b, burried; e, exposed; f, functional; s, structural

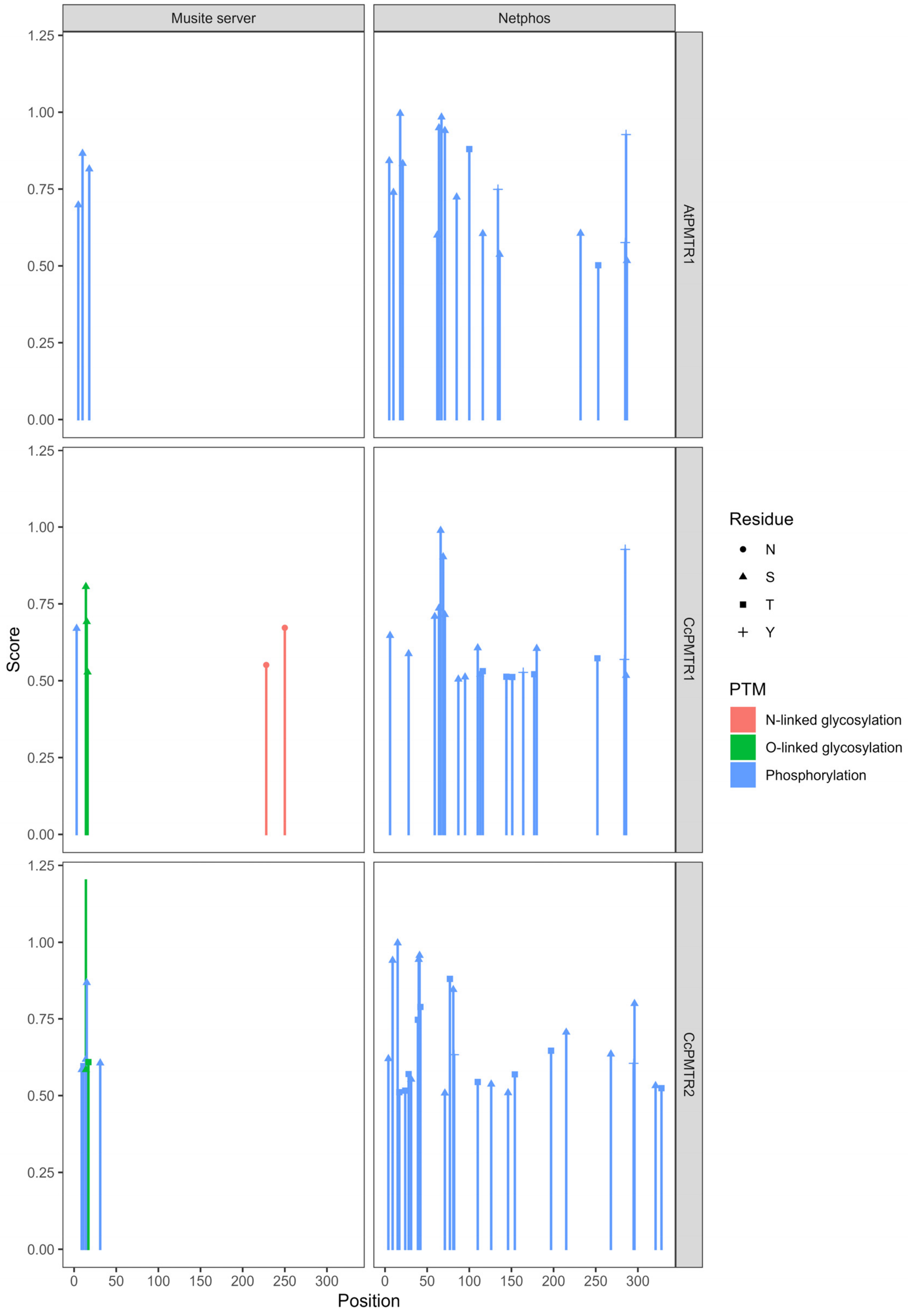

Figure S3: Predicted post-traslational modifications (PTMs) for APMTR1 and C. chinense PMTRs. PTMs were predicted using Musite and NetPhos web servers.

Vertical lines indicate the prediction score for each PTM at a given position, with the symbol at the top denoting the target residue and the color indicating the PTM type

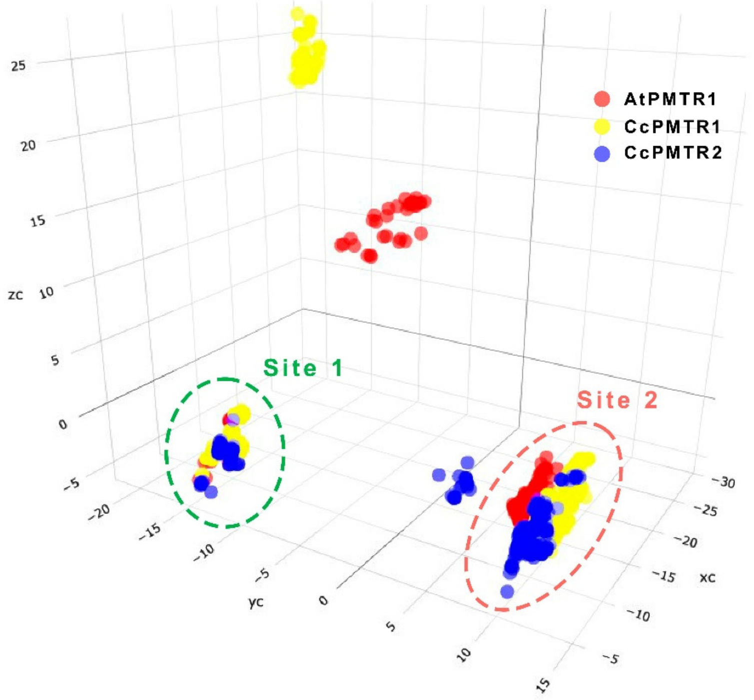

Figure S4: Tri-dimensional distribution of melatonin poses in pPMTRs. An empirical approach was used to predict the most probable binding sites for melatonin, where blind docking simulations were repeated 1000 times for each receptor. The top ranked pose, corresponding to the lowest score, was selected for each simulation. Then the center of mass of the ligand was calculated, and each set of points was classified using a density-based spatial clustering algorithm. The top 3 ranking clusters, those with more points for each receptor, were plotted in a tri-dimensional space. Clusters shared by all three receptors are highlighted as site 1 (green dashed lines) and site 2 (red dashed lines).

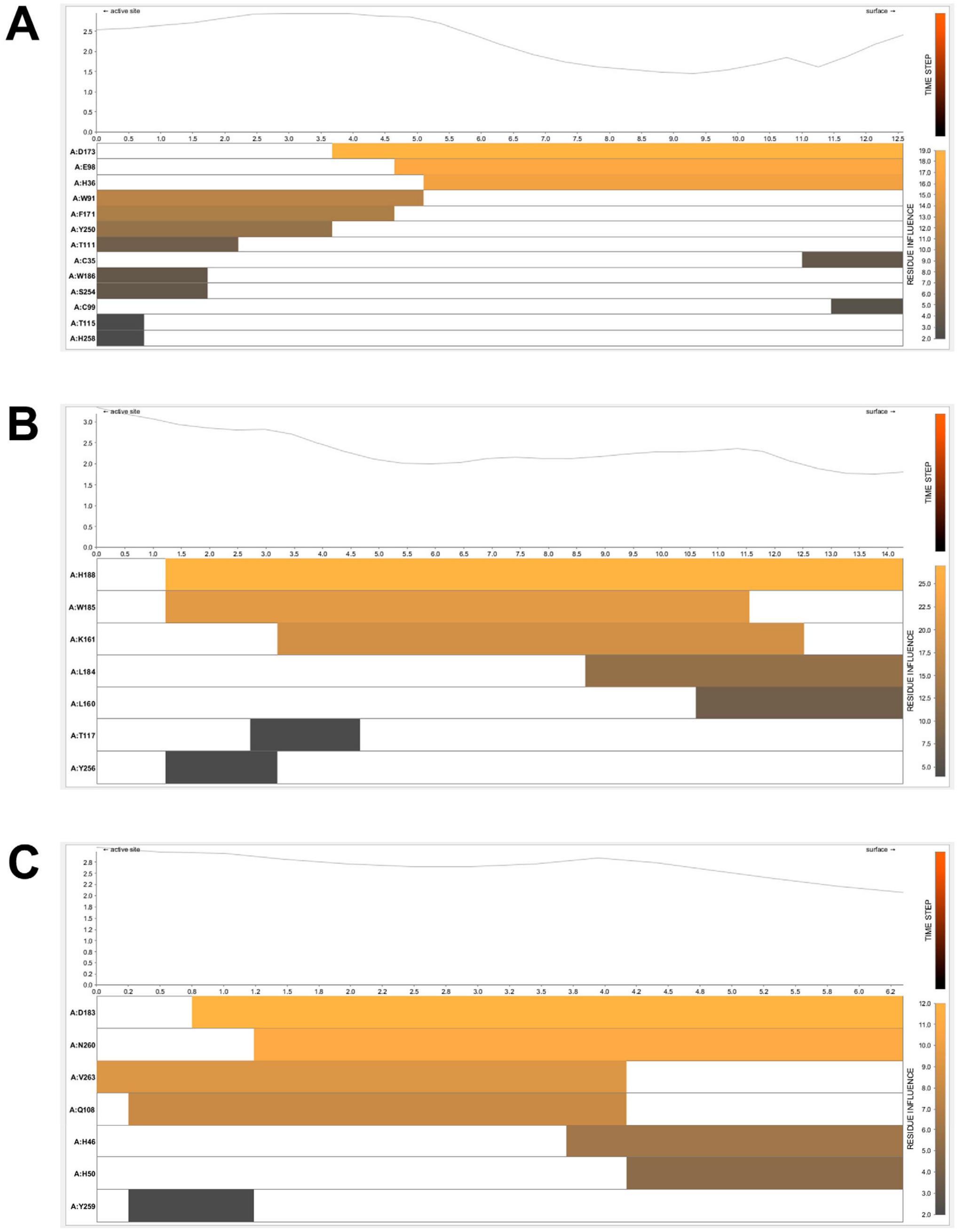

Figure S5: Influential residues to melatonin trajectory through the tunnel accessing the ligand-binding site in pPMTRs. Superior panel indicates the radius variation across the tunnel from the binding site to the receptor surface. Inferior panel highlights residues interacting with the ligand along the tunnel. A, AtPMTR1; B, CcPMTR1; C, CcPMTR2

Bibliography

Omasits, Ulrich, Christian H. Ahrens, Sebastian Müller, and Bernd Wollscheid. 2014. “Protter: Interactive Protein Feature Visualization and Integration with Experimental Proteomic Data.” *Bioinformatics* 30 (6): 884–86. https://doi.org/10.1093/bioinformatics/btt607.

Pupko, Tal, Rachel E. Bell, Itay Mayrose, Fabian Glaser, and Nir Ben-Tal. 2002. “Rate4Site: An Algorithmic Tool for the Identification of Functional Regions in Proteins by Surface Mapping of Evolutionary Determinants Within Their Homologues.” *Bioinformatics* 18 (suppl_1): S71–77. https://doi.org/10.1093/bioinformatics/18.suppl_1.S71.
